# Supplementary material for: Patient satisfaction with the quality of care received is associated with adherence to antidepressant medications
Source: PLoS One. 2024 Jan 5;19(1):e0296062. doi: 10.1371/journal.pone.0296062 (PMC10769059; doi:10.1371/journal.pone.0296062)
Supplement: Attachment — Submitted filename: Response to Reviewers.docx [file pone.0296062.s002.docx]

**Response to Reviewers**

Thank you for the second opportunity to further revise our manuscript. We are most grateful to the reviewers for their very constructive additional comments. We have fully addressed the comments below:

Reviewer #2: Dear Authors, The submitted revision addresses majority of issues mentioned in my primary review. There are only two elements which should be improved, in my opinion. These are:

1) The Kessler Psychological Distress Scale (K6) and the two-item Patient Health Questionnaire depression screener (PHQ-2) used to assess physical and mental health. The explanation was provided in the 'Authors' response', this information, however, is still not available to the readers. I suggest adding it under Methods.

**Response: We agree that adding this information to the manuscript has improved the clarity for how these variables were measured. We have added a description for the measurement of these variables on page #7.**

2) The similar comment is for data collection strategy used for poverty level. Add, please, that it was 'as declared by the respondent'

**Response: Similar to our response above, we have added a description for the measurement poverty status on page #7.**
